# Supplementary figures and images for: Development of a homogeneous time-resolved FRET (HTRF) assay for the quantification of Shiga toxin 2 produced by E. coli
Source: PeerJ. 2021 Jul 28;9:e11871. doi: 10.7717/peerj.11871 (PMC8325423; doi:10.7717/peerj.11871)

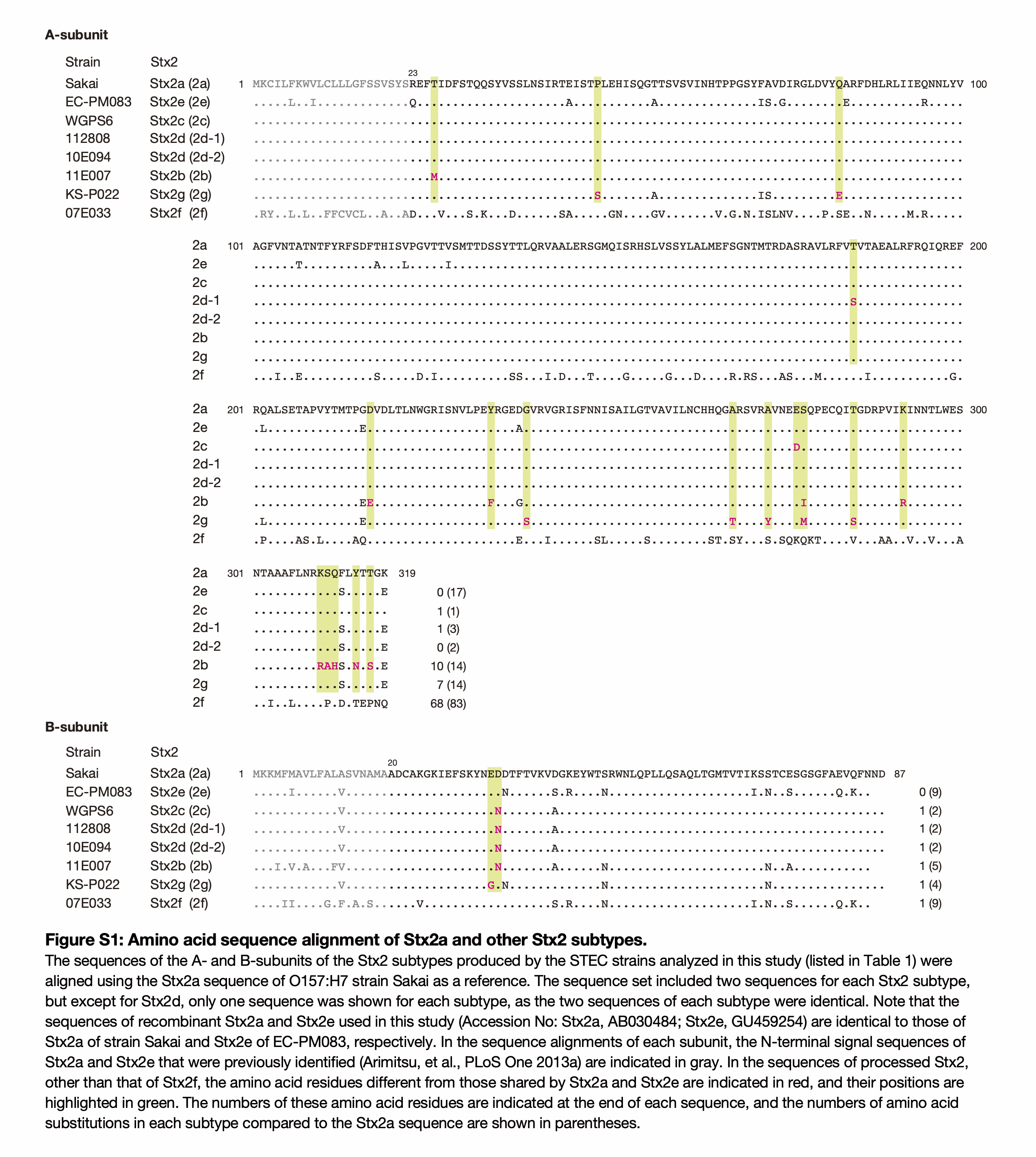

Supplement: Supplemental Information 1 — The sequences of the A- and B-subunits of the Stx2 subtypes produced by the STEC strains analyzed in this study (listed in Table 1) were aligned using the Stx2a sequence of O157:H7 strain Sakai as a reference. The sequence set included two sequences for each Stx2 subtype, but except for Stx2d, only one sequence was shown for each subtype, as the two sequences of each subtype were identical. Note that the sequences of recombinant Stx2a and Stx2e used in this study (Accession No: Stx2a, AB030484; Stx2e, GU459254) are identical to those of Stx2a of strain Sakai and Stx2e of EC-PM083, respectively. In the sequence alignments of each subunit, the N-terminal signal sequences of Stx2a and Stx2e that were previously identified (Arimitsu, et al., PLoS One 2013a) are indicated in gray. In the sequences of processed Stx2, other than that of Stx2f, the amino acid residues different from those shared by Stx2a and Stx2e are indicated in red, and their positions are highlighted in green. The numbers of these amino acid residues are indicated at the end of each sequence, and the numbers of amino acid substitutions in each subtype compared to the Stx2a sequence are shown in parentheses. [file peerj-09-11871-s001.jpg]

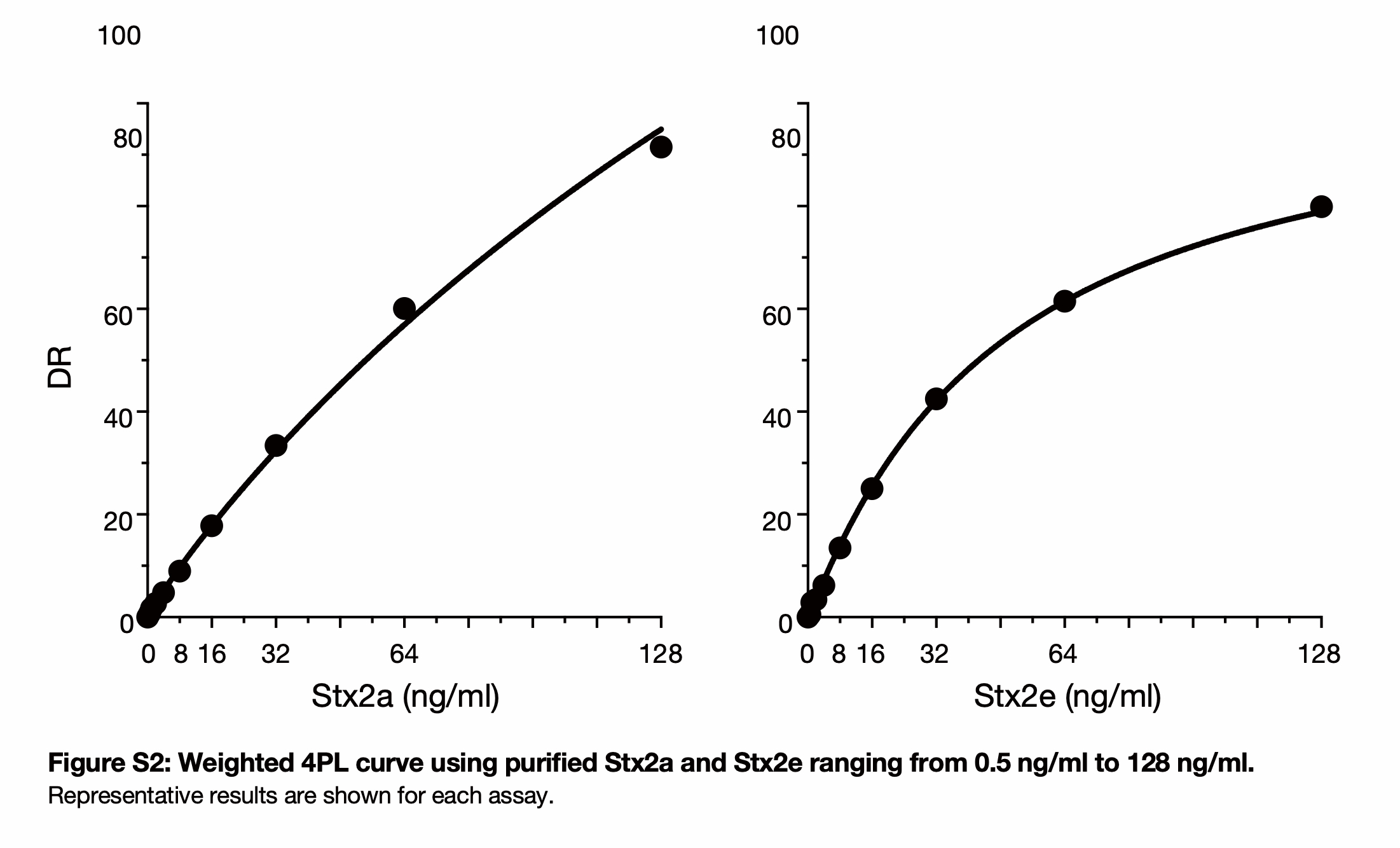

Supplement: Supplemental Information 2 — Representative results are shown for each assay. [file peerj-09-11871-s002.jpg]
