# Supplementary material for: Development of a homogeneous time-resolved FRET (HTRF) assay for the quantification of Shiga toxin 2 produced by E. coli
Source: PeerJ. 2021 Jul 28;9:e11871. doi: 10.7717/peerj.11871 (PMC8325423; doi:10.7717/peerj.11871)
Supplement: Supplemental Information 3 — The mean value of buffer control was 15.3 (Exp. 1) or 23.0 (Exp. 2). [file peerj-09-11871-s003.pdf]

Table S1. The detection limit of Stx2a and the comparison of signal intensities obtained by two series of experiments using different ranges of toxin concentration (three independent measurements were performed at each toxin concentration in each series of experiment).

	Delta ratio (DR)				
	1st.	2nd.	3rd.	average	SD
Exp.1 (0.25-256 ng/ml)					
0.25	0.0	0.6	0.2	0.3	0.3
0.5	0.7	0.8	0.6	0.7	0.1
1.0	1.7	2.1	1.0	1.6	0.6
2.0	2.7	3.6	2.0	2.8	0.8
4.0	4.8	5.2	3.9	4.6	0.7
8.0	9.0	9.8	8.3	9.0	0.8
16	17.8	17.9	16.1	17.3	1.0
32	33.4	33.1	30.0	32.2	1.9
64	60.1	56.8	52.1	56.3	4.0
128	91.5	81.7	76.1	83.1	7.8
256	97.9	87.1	83.2	89.4	7.6
Exp. 2 (1.0-64 ng/ml)					
1.0	2.2	2.3	1.3	1.9	0.6
2.0	4.2	4.2	3.3	3.9	0.5
4.0	7.4	6.8	6.8	7.0	0.4
8.0	13.5	14.3	14.3	14.0	0.5
16	26.5	26.9	26.2	26.5	0.3
32	49.0	50.7	48.6	49.4	1.1
64	87.6	86.4	86.5	86.8	0.7

The mean value of buffer control was 15.3 (Exp. 1) or 23.0 (Exp. 2).
