# Supplementary material for: Development of a homogeneous time-resolved FRET (HTRF) assay for the quantification of Shiga toxin 2 produced by E. coli
Source: PeerJ. 2021 Jul 28;9:e11871. doi: 10.7717/peerj.11871 (PMC8325423; doi:10.7717/peerj.11871)
Supplement: Supplemental Information 4 [file peerj-09-11871-s004.pdf]

Table S2. Signal intensities of Stx2e, Stx2f and Stx1a in the HTRF assay.

Purified Stx (ng/ml)	Delta ratio (DR)		
	1st.	2nd.	3rd.
Stx2e			
0.25	0.2	0.3	0.8
0.5	0.5	0.8	0.9
1.0	2.9	2.4	3.0
Stx2f			
320	-1.2	0.0	1.5
640	-0.9	0.2	1.1
1280	-0.5	1.7	2.0
Stx1a			
320	0.9	0.5	0.4
640	0.5	1.2	0.4
1280	1.2	0.8	0.6
